# Erythema Ab Igne in a 12-Year-Old Boy Diagnosed via Telemedicine

**DOI:** 10.7759/cureus.11577

**Published:** 2020-11-19

**Authors:** Timothy R Nield, Nancy E Brunner, Zachary Zinn

**Affiliations:** 1 Department of Pediatrics, West Virginia University School of Medicine, Morgantown, USA; 2 Department of Dermatology, West Virginia University School of Medicine, Morgantown, USA

**Keywords:** erythema ab igne, livido reticularis

## Abstract

In March of 2020, an otherwise healthy 12-year-old boy developed a unilateral patch of reticulated erythema limited to his left lower extremity. The child could not be examined in the clinic due to limited in-person appointments during the severe acute respiratory syndrome coronavirus 2 (SARS-CoV-2) pandemic, so he was examined via a telemedicine visit. The diagnosis of erythema ab igne was made as his mother verified that the child was spending approximately two hours per day playing video games in the cold basement of his house, with a space heater positioned close to his left leg. Our case of erythema ab igne is unique due to the relatively young age of the affected child, and it provides an example of how this diagnosis can be made via a telemedicine visit. Being able to recognize the classic appearance of erythema ab igne through the scrutiny of photographs and obtaining pertinent history can preclude the need for an in-person visit during times when home sequestration may be a necessity.

## Introduction

Erythema ab igne presents as a net-like macular discoloration due to chronic exposure to a heating source. The word “igne” is derived from the Latin word for “fire,” and therefore, the translation of erythema ab igne is “redness by fire” [1].

Cases of erythema ab igne secondary to patients being exposed to a fire for prolonged timeframes were published in the medical literature in 1911 [2,3]. Following the introduction of central heating into most households, the occurrence of erythema ab igne has become uncommon. In more recent times, a variety of stimuli have been described as causing erythema ab igne. Samaan et al. [4] described three cases in patients with sickle cell anemia, aged 17 years to 19 years, following the use of heating pads applied to the skin of their flank and/or abdomen to relieve pain. Reports of erythema ab igne associated with the heat generated from a laptop pressed against the chest or thighs, including the 12-year-old patient of Arnold and Itin [5], have been described. Additionally, Dessinioti et al. [6] described three patients with anorexia nervosa aged 15 years to 17 years who presented with erythema ab igne associated with frequent sitting in front of heat radiators during cold weather months. We present a case of erythema ab igne in a 12-year-old boy resulting from prolonged exposure to a portable heating device during home sequestration, diagnosed via telemedicine.

## Case presentation

**Figure 1:**
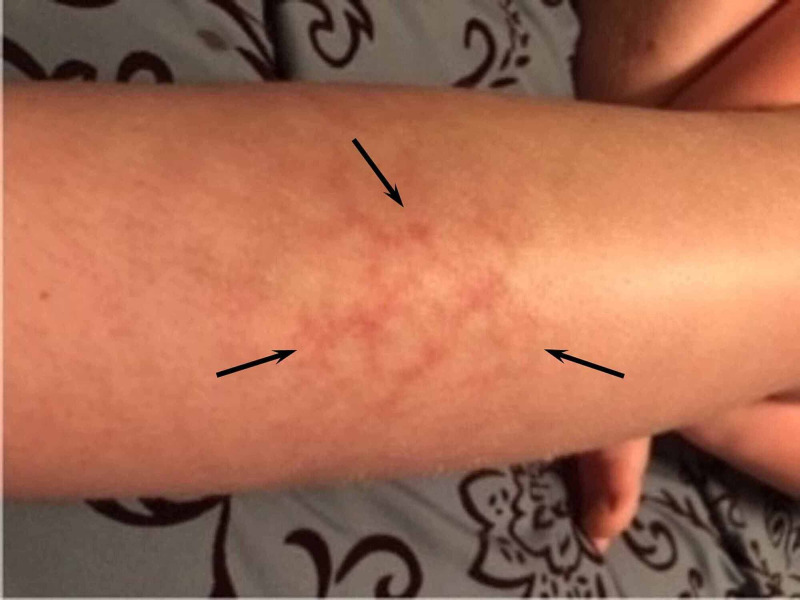
Reticular erythema of the shin of the 12-year-old patient

During home sequestration enacted due to the severe acute respiratory syndrome coronavirus 2 (SARS-CoV-2) pandemic in March of 2020, an otherwise healthy 12-year-old boy developed a unilateral asymptomatic rash limited to his left lower extremity. Household contacts lacked a similar rash. Because of the pandemic and unseasonably cold weather, he had not traveled nor left his house in several days. The boy had no history of fever, malaise, insect exposure, nor prolonged compression or trauma to the affected extremity. Since this occurred during the upswing of the pandemic, the child could not be seen in-person in the clinic, and he was examined via a telemedicine visit. The child appeared well on the computer screen with clear conjunctiva and mucus membranes. No skin lesions were seen, except for an area of reticulated erythema on his left shin (Figure 1).

**Figure 2:**
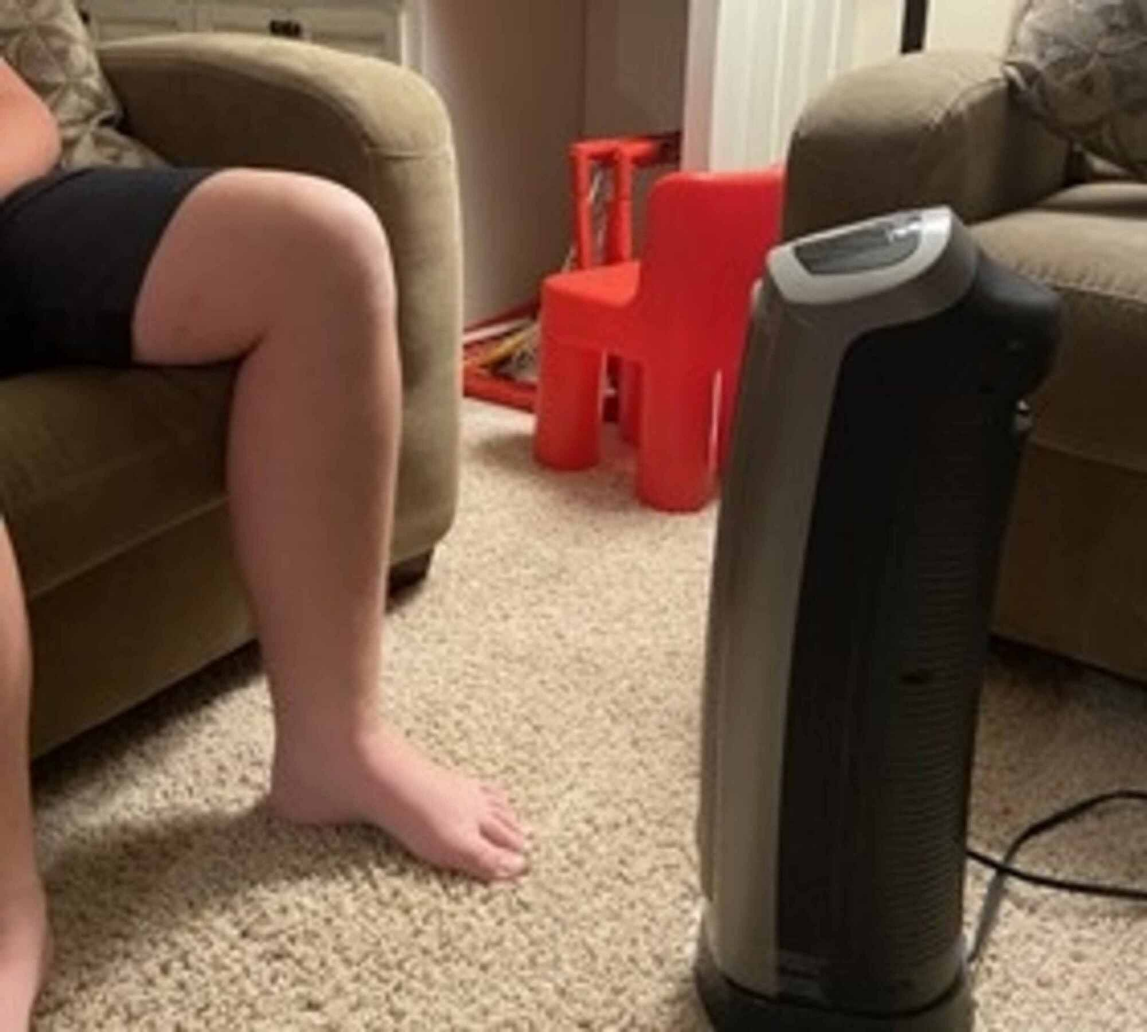
Exposure of the left shin to the portable heater

Due to the reticular pattern of the rash, a diagnosis of parvovirus infection was initially considered, even though there were no systemic symptoms, prior “slapped cheek” appearance, or generalized distribution of the rash. Since the diagnosis was in doubt and further in-person evaluation could not be performed, such as obtaining parvovirus titers, a pediatric dermatologist was consulted. An additional historical question was posed about the child’s potential chronic exposure to a heater. The mother verified that since the boy had been sequestered in his home for several days due to the pandemic, he was spending approximately two hours per day sitting on a couch playing video games in the cold basement of his house, with a space heater positioned close to his left leg (Figure 2).

Due to the history of exposure to a portable heater and the classic appearance of the rash, the clinical diagnosis of erythema ab igne was made. The patient was advised to avoid direct exposure to the portable heater, and reassurance was provided that no other intervention was required except for follow-up examination to ensure resolution of the rash. After approximately four weeks of avoidance of the direct heating source, the skin erythema was much reduced. The child remained otherwise asymptomatic.

## Discussion

Our case of erythema ab igne is unique due to the relatively young age of the affected child [5,7]. It provides an example of how this diagnosis can be made via a telemedicine visit. Obtaining a thorough history, including an inquiry about frequent exposure to a heating source, such as a fire, radiator, heating pad, car heater, heated chair, hot water bottle, stove, and laptop computer [8] and being able to recognize the classic appearance of the rash are crucial components to making the diagnosis. Riahi and Cohen’s literature review includes 15 reports of erythema ab igne secondary to skin exposure to a laptop computer in patients with a mean age of 25 years [9]. The thighs are the most common bodily site affected, but the breasts may also be involved if the laptop rests on the chest. Consultation with a dermatologist should be sought if the diagnosis is in doubt.

Treatment of erythema ab igne includes removal of exposure to the heat source and follow-up observation to ensure resolution of the rash. Prevention of erythema ab igne is possible through avoidance of prolonged heat exposure. For example, heat exposure from laptop computers can be avoided by incorporating practical measures such as the placement of a barrier pad between the laptop and the thighs [10].

Clinicians should be familiar with rashes that may be confused with erythema ab igne. Livedo reticularis also presents as a net-like macular discoloration, vascular appearing, and typically found diffusely in infants and on the lower extremities of adults. Underlying systemic disorders may or may not be associated with a diagnosis of livedo reticularis. In contrast to erythema ab igne, livedo reticularis typically appears with cold exposure and may resolve within minutes of rewarming, while erythema ab igne appears with heat exposure and remains chronically if the heating source is not removed.

Aria et al. [11] describe other dermatologic conditions occurring in various age groups that also present with a similar reticulated pattern to erythema ab igne. Examples include cutis marmorata and livedo racemosa. Cutis marmorata is a benign diffuse mottling of the skin most commonly described in neonates, which occurs during exposure to cold environments and improves with rewarming. Livedo racemosa is an irregular violaceous discoloration of the limbs or more widespread on the body of adolescents and adults and is associated with underlying systemic disorders. It is prudent for clinicians also to be aware of other dermatologic disorders that can result from thermal exposure, such as basal cell carcinoma and burns, as described in the review by Forouzan et al. [12].

## Conclusions

Varied circumstances may force children and their families to remain indoors for prolonged periods. If individuals spend more time indoors in front of heating sources, increased episodes of erythema ab igne or other thermal injuries may occur. Circumstances may also arise that limit in-person clinic appointments. Primary care clinicians’ abilities to recognize the classic appearance of erythema ab igne through a telemedicine visit or the scrutiny of photographs after obtaining pertinent history can preclude the need for an in-person visit and the need for consultation with a specialist.
